# Small Finger Proximal Interphalangeal Joint Chronic Arthritis Secondary to Leprosy: A Case Report

**DOI:** 10.7759/cureus.36915

**Published:** 2023-03-30

**Authors:** Ahmed M Elbayer, Mohamed M Ibrahim, Sara Alharami, Iqbal Wani, Ahmed H Elhessy

**Affiliations:** 1 Plastic and Reconstructive Surgery, University of Tennessee, Memphis, USA; 2 Department of Medicine, University of Maryland School of Medicine, Baltimore, USA; 3 Department of Plastic Surgery, Hamad General Hospital, Doha, QAT; 4 Orthopedics, The Rubin Institute for Advanced Orthopedics/Sinai Hospital, Baltimore, USA

**Keywords:** arthropathy, proximal interphalangeal joint, hand, hansen’s disease, leprosy

## Abstract

Leprosy (Hansen's disease) is a multisystem, chronic infectious disease that still exists. It is caused by *Mycobacterium leprae*. Musculoskeletal features are non-consistent and can lead to misdiagnosis and mistreatment. We report the case of a 23-year-old male with the right small finger (RSF) proximal interphalangeal (PIP) joint arthropathy related to leprosy. This was his first encounter with seeking medical advice regarding his condition. The patient was diagnosed and treated with surgical debridement, volar plate arthroplasty for the affected proximal interphalangeal joint, and the recommended multi-drug therapy regimen. The pathological effects of leprosy on the bones and joints have been attributed to several theories, with peripheral nerve neuropathy being the primary cause. Early detection of leprosy is crucial for effective management, preventing further disease transmission, and minimizing the risk of developing complications.

## Introduction

Leprosy (Hansen's disease) is a multisystem, chronic infectious disease caused by *Mycobacterium leprae* [1-4]. It primarily affects the skin, the peripheral nerves, and the musculoskeletal system. Leprosy can cause muscle weakness and wasting, nerve damage, joint deformities, bone infections, and immune-mediated reactions that affect the musculoskeletal system. Common joint deformities include claw hand and foot drop [5-9]. Musculoskeletal features sometimes constitute the initial presentation or mimic other connective tissue disorders, which can lead to misdiagnosis and mistreatment [8].

We present the case of a 23-year-old male who presented to the emergency department with chronic arthritis and abundant *M. leprae* in his right small finger (RSF) proximal interphalangeal (PIP) joint. The case was successfully managed with surgical debridement, volar plate arthroplasty, and a multi-drug therapy regimen.

## Case presentation

**Figure 1:**
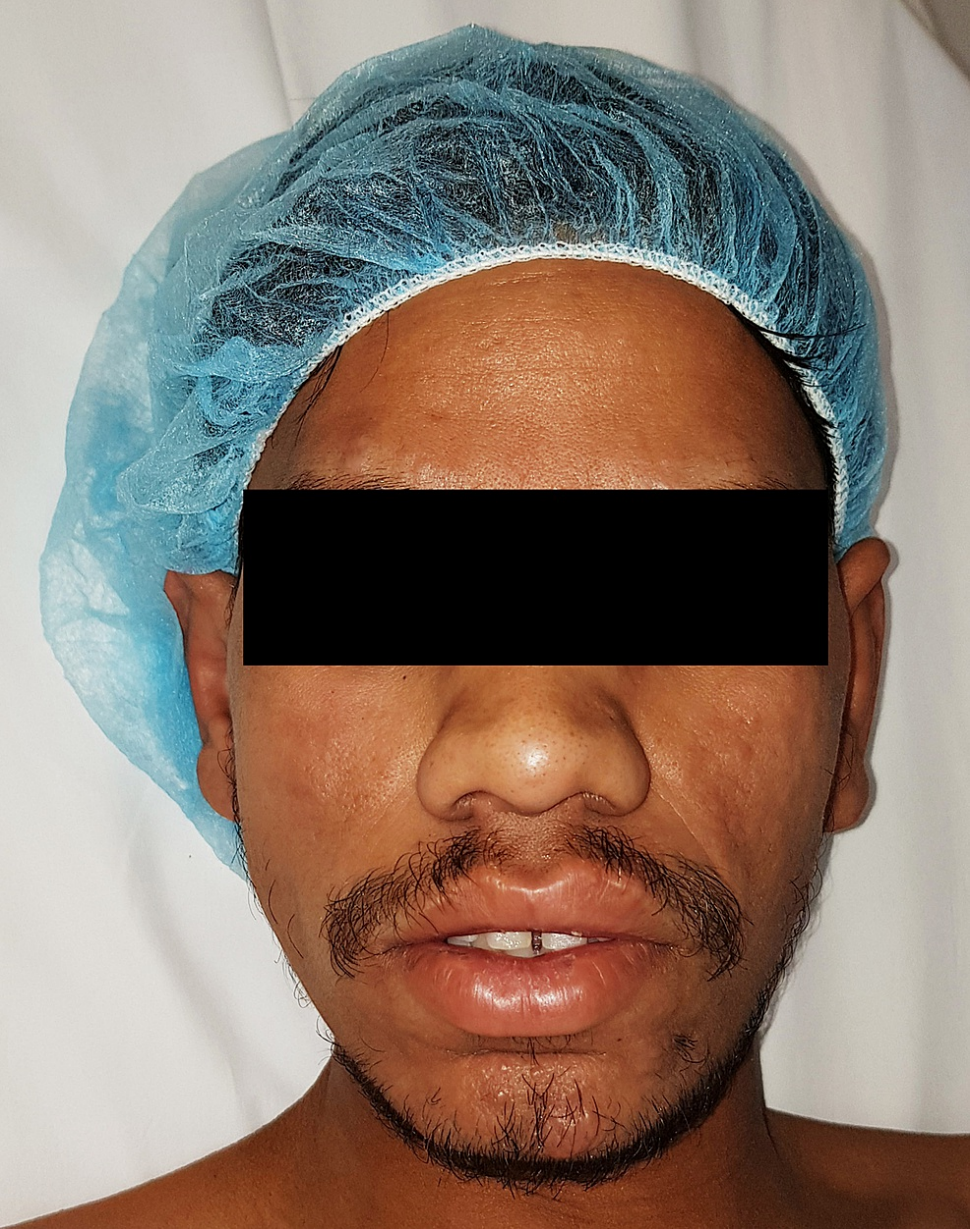
Physical examination revealed some facial changes, including prominent convexities and furrowed creases, anteroposterior view.

**Figure 2:**
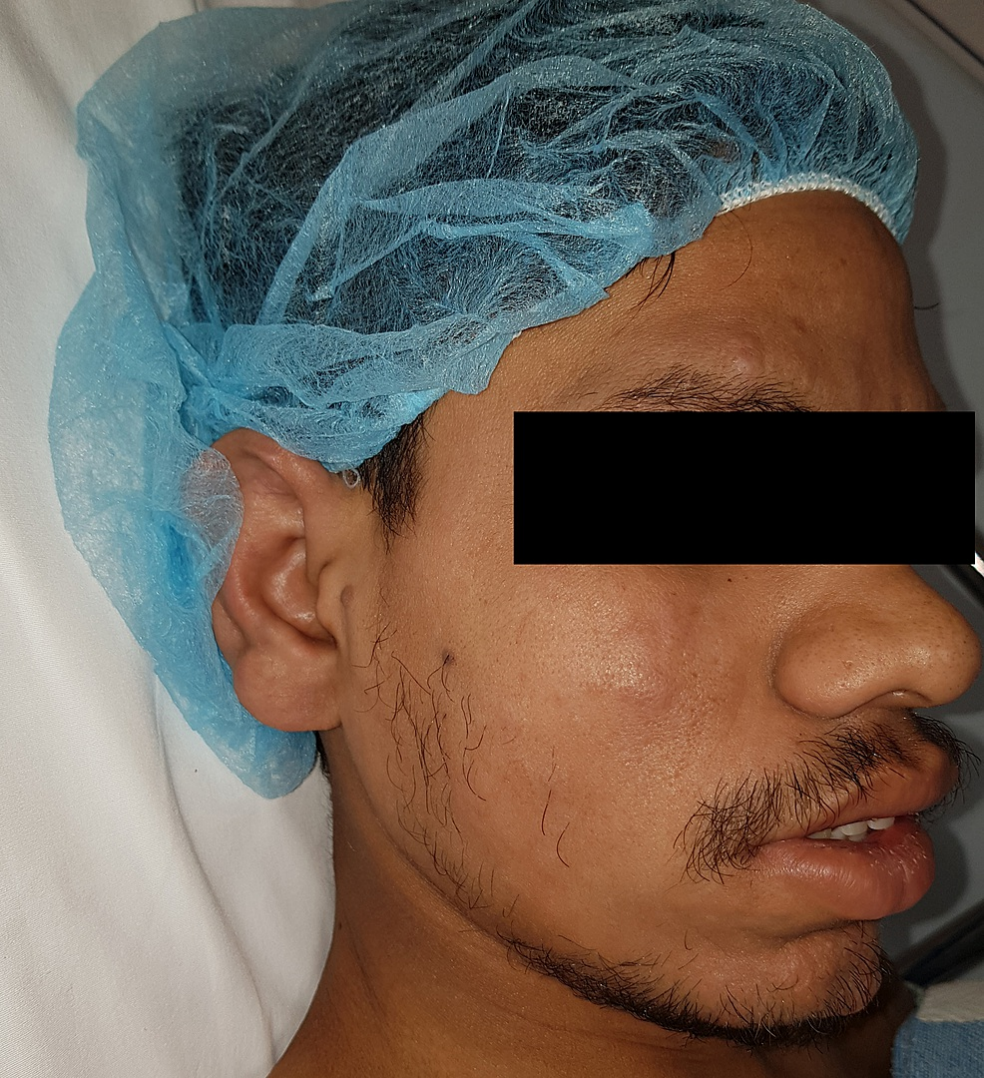
Facial changes from an oblique view.

**Figure 3:**
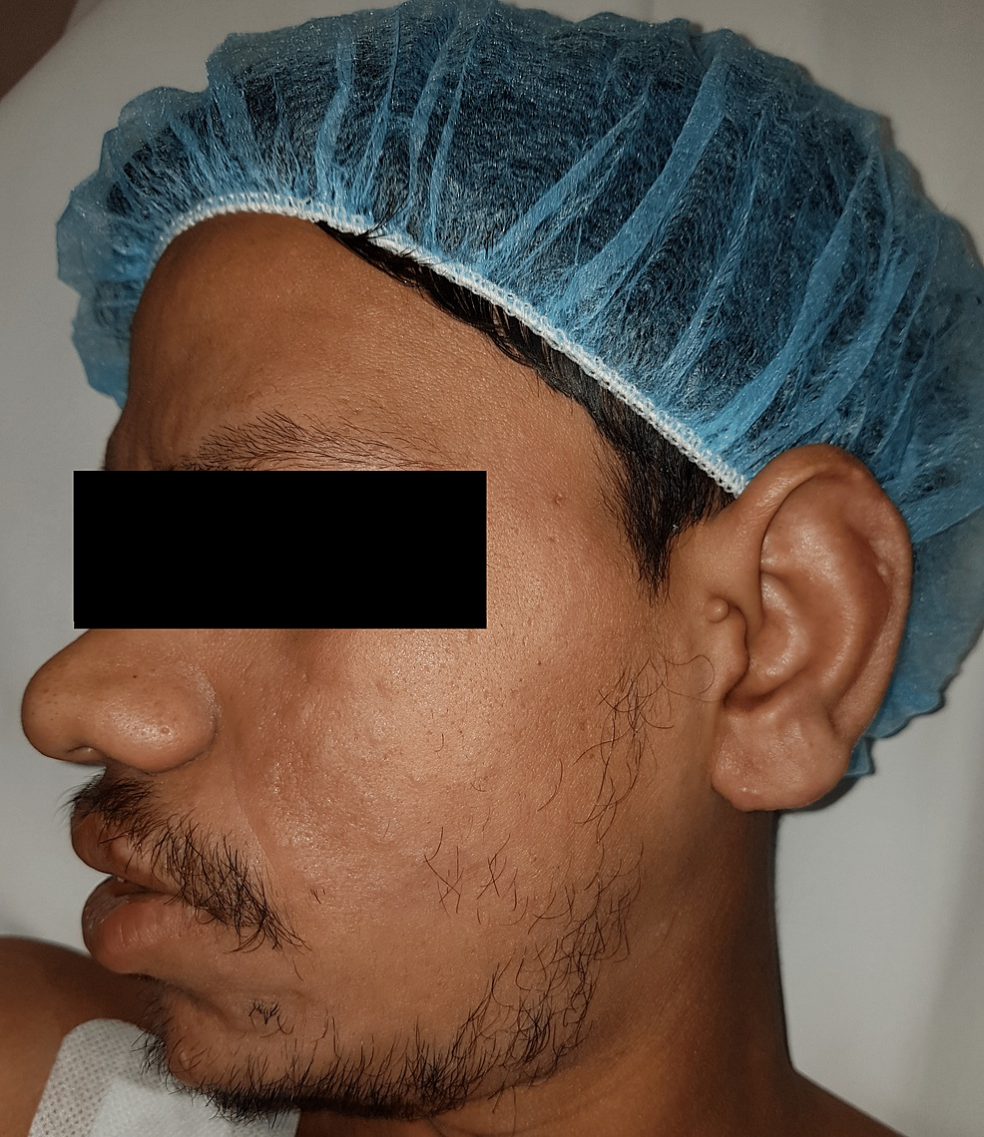
Facial changes from a left lateral view.

A 23-year-old male from the far east presented to the emergency department with swelling of his RSF for several months. The patient had a long history of deformity, shortening, and stiffness of his RSF over the past three years. He has also complained of some limitations in his activities of daily living. Additionally, the patient reported noticeable facial changes during the same period. Physical examination revealed facial changes, including prominent convexities and furrowed creases (Figures 1-3).

**Figure 4:**
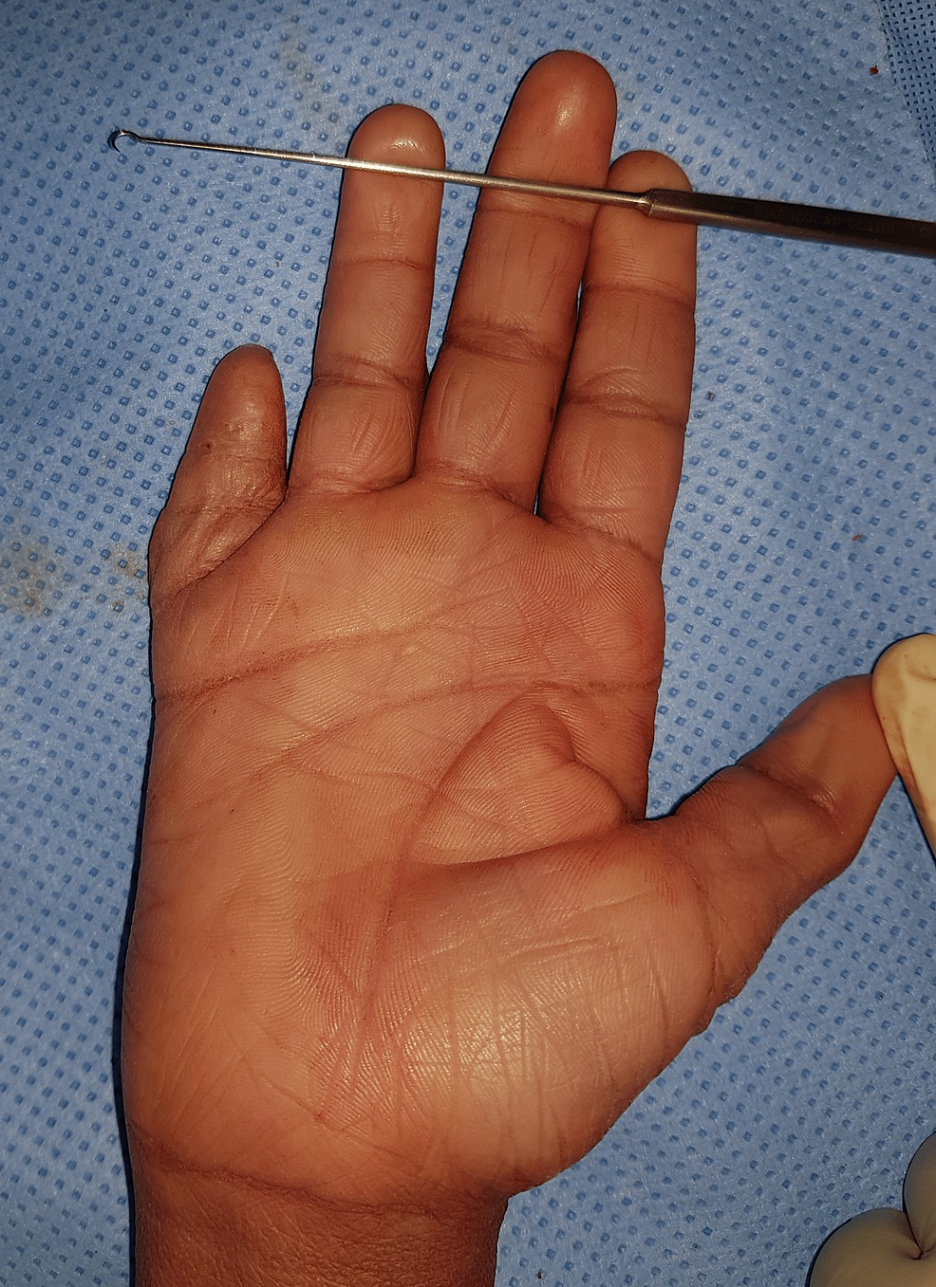
Right-hand examination revealed RSF shortening and a fixed flexion deformity of the RSF-PIP joint. RSF: right small finger, PIP: proximal interphalangeal.

A right-hand examination revealed RSF shortening and a fixed flexion deformity of the RSF-PIP joint (Figure 4).

**Figure 5:**
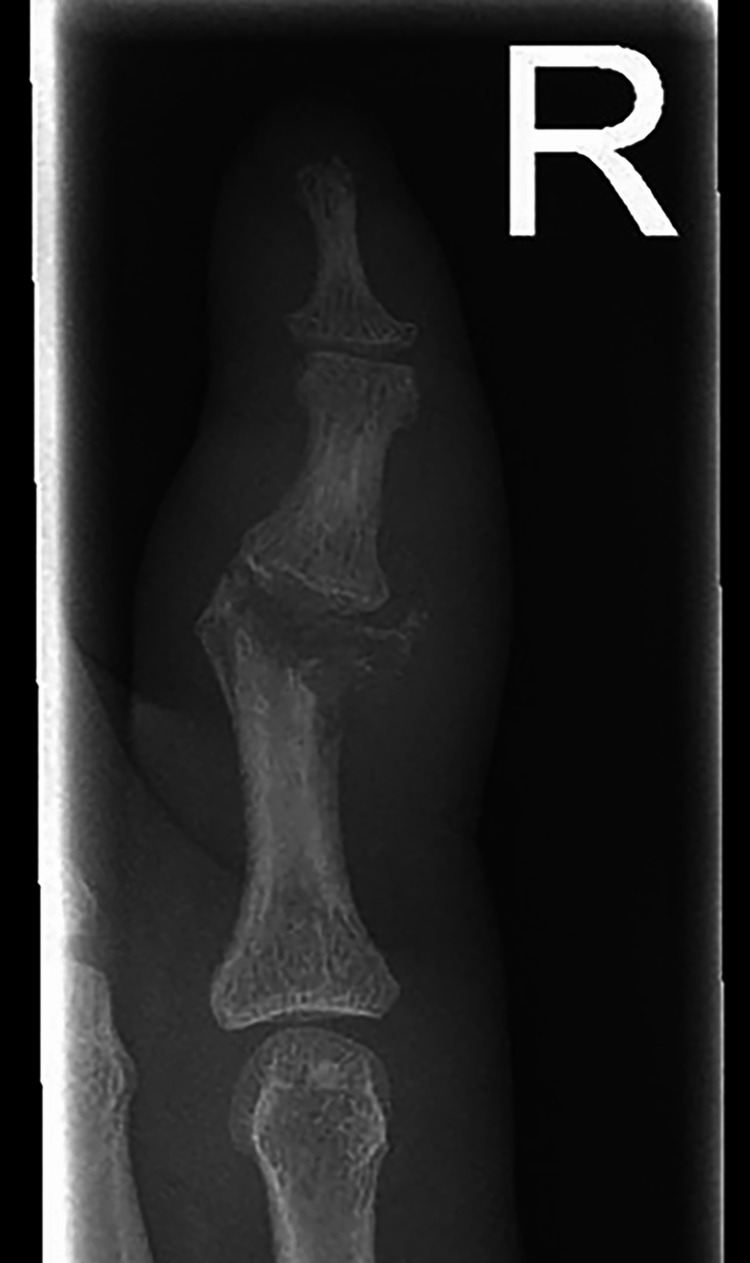
Plain radiographs showed RSF-PIP joint destruction with osteolysis and an increased soft tissue shadow, AP view. RSF: right small finger, PIP: proximal interphalangeal, AP: anteroposterior view.

**Figure 6:**
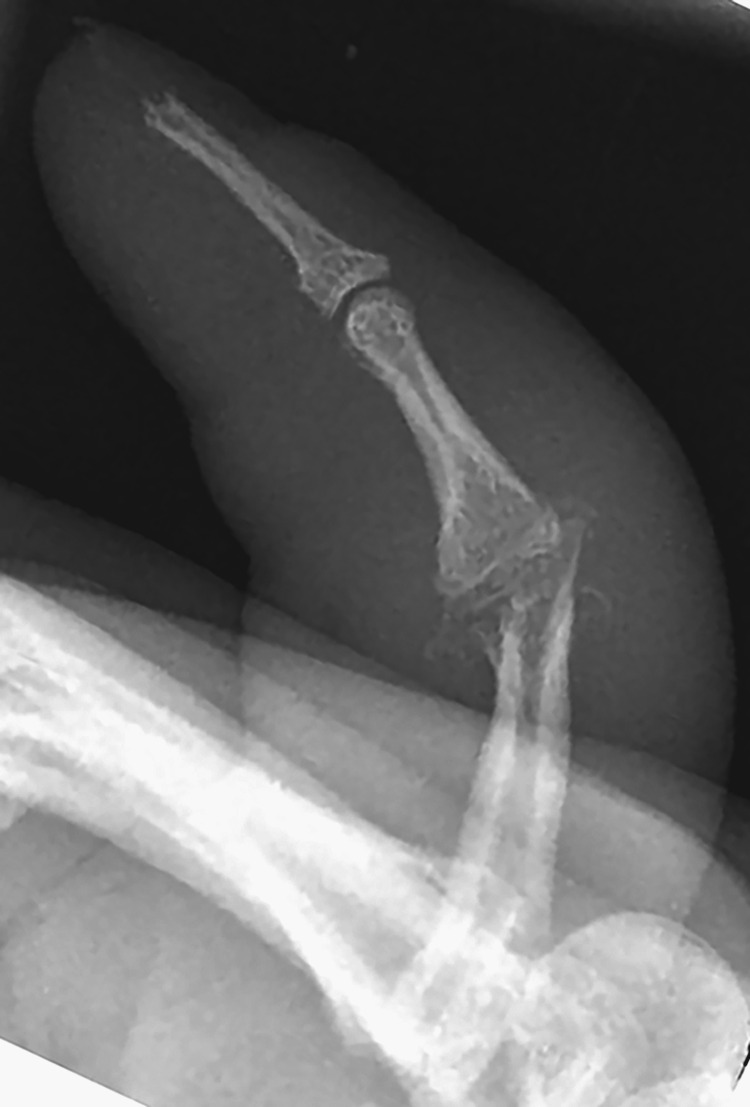
Plain radiographs showed RSF-PIP joint destruction with osteolysis and an increased soft tissue shadow, lateral view. RSF: right small finger, PIP: proximal interphalangeal.

At the same time, a neurovascular examination revealed hyperesthesia along the ulnar nerve distribution of his hand, including the RSF. Plain radiographs showed RSF-PIP joint destruction with osteolysis and an increased soft tissue shadow (Figures 5-6). The initial differential diagnosis of the finger lesion included a local tumor, osteomyelitis, a soft tissue granuloma, an infection, gout, and arthritis. The patient demonstrated no systemic manifestations of bacterial infection (no fevers or chills), and laboratory findings were unremarkable, with a white blood cell count of 4.1 × 109/L. The infectious disease team was consulted, and leprosy was included as a potential differential diagnosis given the clinical presentation and features of the patient, including hyperesthesia, skin lesions, joint swelling, peripheral neuropathy, and leonine facies. Given that leprosy has negative serological markers and a biopsy is needed, we have decided to proceed surgically to remove the RSF joint lesion and send for histopathological analysis.

**Figure 7:**
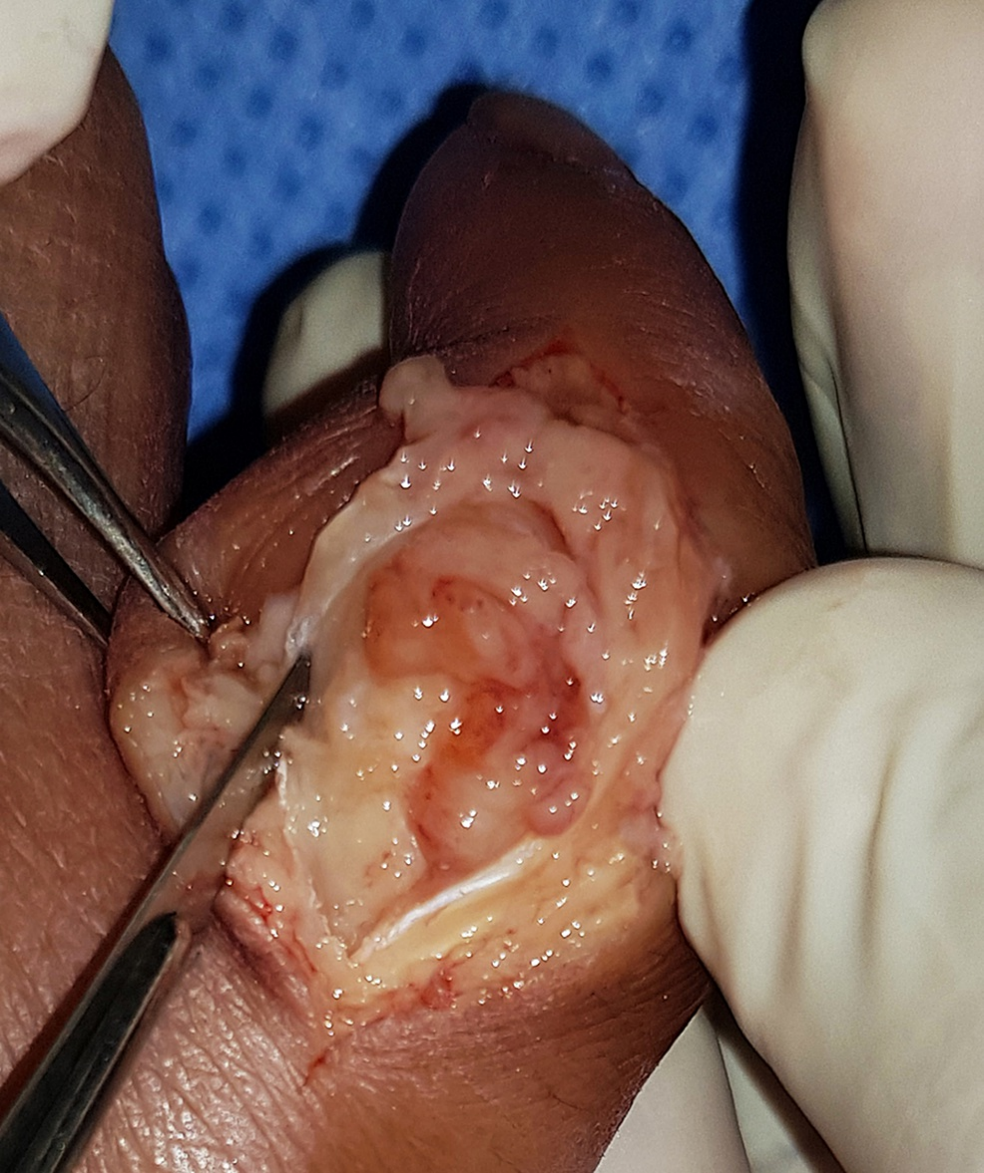
Intraoperative clinical photograph showing RSF-PIP joint destruction. RSF; right small finger, PIP; proximal interphalangeal joint.

Briefly, under local anesthesia, a dorsal longitudinal incision was centered over the RSF-PIP joint. The PIP joint was filled with serous discharge, and the articular surface was completely destroyed (Figure 7).

A biopsy of the granuloma was sent for histopathological examination together with a 3-mm punch biopsy from the forehead. Joint debridement was completed and followed by volar plate arthroplasty by releasing the volar plate transversally on the proximal origin. Followed by transposing the volar plate's released ends posteriorly and suturing it using non-absorbable sutures to the dorsal joint capsule, covering all of the middle phalanx's proximal articular surface.

**Figure 8:**
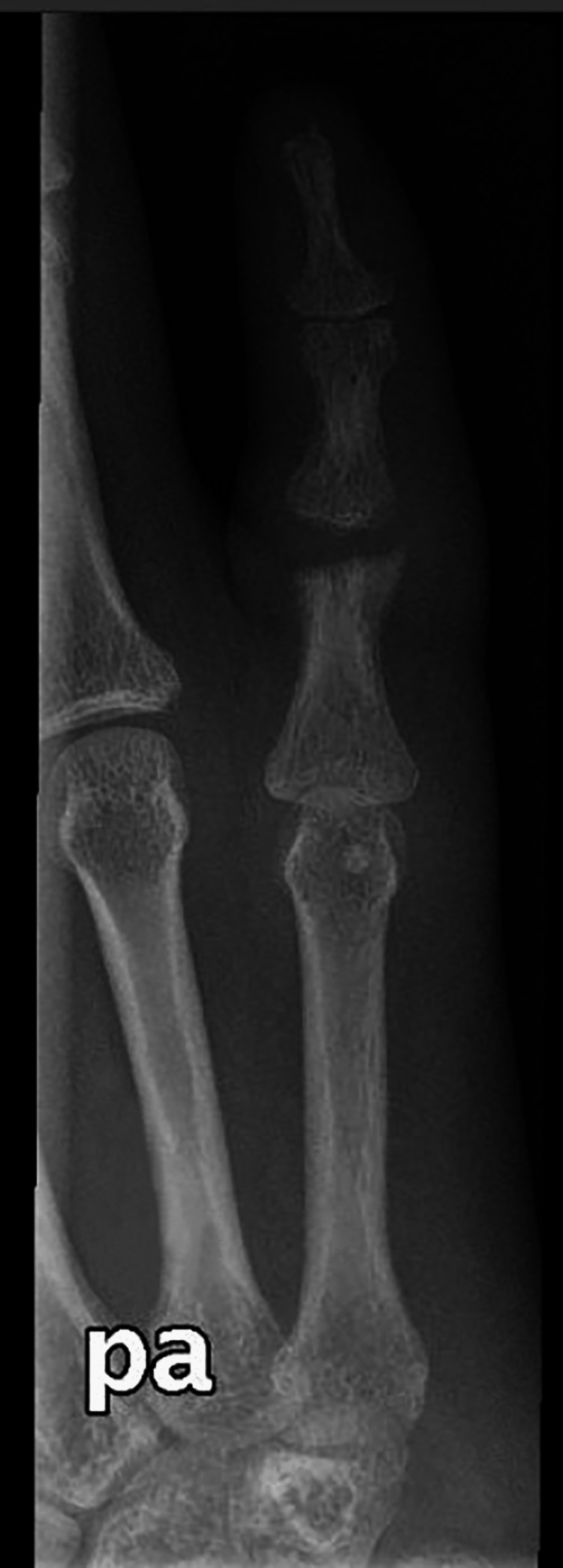
Postoperative radiographs, anteroposterior view.

**Figure 9:**
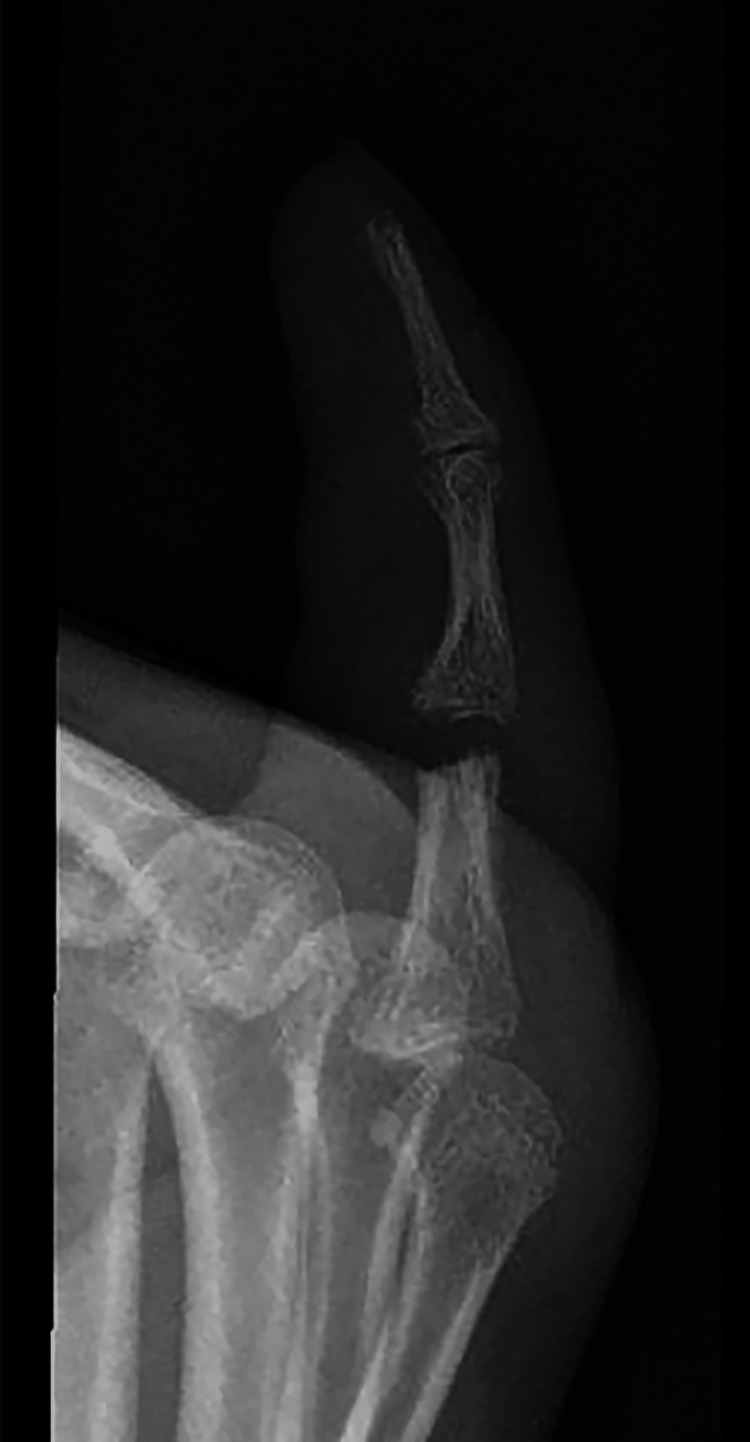
Postoperative radiographs, oblique view.

Postoperative radiographs are shown in Figures 8-9. Histopathological staining with the Wade-Fite Stain was positive, and the diagnosis of lepromatous leprosy was confirmed.

The patient was followed up for a total of 12 months. There was an improvement in his RSF-PIP joint range of motion (10 to 65 degrees) without postoperative therapy. Under the care of the infectious disease department, the patient completed the recommended multi-drug therapy regimen: dapsone 100 mg once daily and rifampin 600 mg daily for a total of 12 months [10].

## Discussion

In 1873, the Norwegian physician Gerhard-Henrik Armauer Hansen published his first report proving that leprosy is an infectious disease, not a curse [10,11]. Despite his discovery, the main treatment option for leprosy was the isolation of the infected in leper colonies [11,12]. As a result, leprosy is known as a neglected tropical disease. However, recent reports show that more than 200,000 newly diagnosed cases occur yearly in more than 120 countries [2]. These reports eliminate the false idea that "leprosy is a vanishing disease."

Musculoskeletal pathological manifestations of leprosy can be attributed to the neuropathic effect of the disease. This process is initiated by *M. leprae* infiltration of the peripheral nerves, which initiates a destructive cascade. This process of peripheral nerve destruction is achieved by a CD4 T-cell-mediated granulomatous process [13]. The residual damage to the affected nerves can be related to the disease's duration, the extent of nerve damage at the time of diagnosis, and the onset and effectiveness of the treatment [13,14].

Hands with arthritis, peripheral neuropathy with thickened nerves, and forearm/hand tenosynovitis should attract attention toward the diagnosis of leprosy. In an explanation of the orthopedic complications of leprosy on bones, Moonot et al. [15] related the pathological effects of leprosy on the joints to multiple factors, including direct infection of the bone and the surrounding soft tissue, trauma, or secondary infection. The damage can also be attributed to the secondary osteoporotic bone changes caused by the immobilization of the fixed contractured fingers. Finally, bone changes in leprosy are primarily related to established neuropathy [13,15]. We believe all these factors played a role in the pathology of our presented case.

Granulomatous reactions on the small bones of the hands and feet characterize bony lesions in leprosy. Radiographically, they are evident as focal areas of bone resorption, destruction, and increased rarefaction. The articular surface may be collapsed, flattened, destructed, or resorbed [15,16]. In our case, there was an apparent deformity, shortening of the RSF, and symptoms of ulnar nerve neuropathy. The plain radiographs showed PIP joint deformity, fracture, osteolysis, and resorption. Intraoperatively, the PIP joint was filled with serous discharge and lepromatous granuloma. Excision of the damaged bone followed by volar plate arthroplasty was the preferred surgical treatment option. Oral antibiotics were administered according to the recent guidelines.

The primary strategy is early detection and prevention of such manifestations using antileprosy drugs. In addition, oral steroids for nerve damage are recommended for patients who develop acute peripheral nerve damage from leprosy [15,17]. Other surgical treatment options have been described for the hand manifestations of leprosy [13]. Physical therapy is essential to limit finger stiffness; this can be achieved by active exercises, passive stretching, and splinting. Avoiding finger stiffness can play an essential role in the decision to undergo tendon transfer procedures [5]. Early nerve decompression can be considered on a case-by-case basis.

## Conclusions

Leprosy is not a vanishing disease. Its musculoskeletal manifestation can be the initial presentation, which can mimic other connective tissue diseases. These symptoms are likely to be overlooked or not always appreciated, which can lead to misdiagnosis and mistreatment. Most of the manifestations observed in the small joints of the hands are related to the long-standing neuropathic effect of the disease. The primary treatment is focused on the early detection and prevention of such complications by using antileprosy drugs. Attention should be paid to diagnosing musculoskeletal injuries related to leprosy and how to treat them.
